# Knowledge and practice of iodized salt utilization among reproductive women in Addis Ababa City

**DOI:** 10.1186/s13104-018-3847-y

**Published:** 2018-10-16

**Authors:** Meseret Mamo Bazezew, Walelegn Worku Yallew, Aysheshim kassahun Belew

**Affiliations:** 1 Development and Research Institute, Addis Ababa, Ethiopia; 2 Department of Environmental, Occupational Health and Safety, Institute of Public Health, University of Gondar, Gondar, Ethiopia; 3 Department of Human Nutrition, Institute of Public Health, University of Gondar, Gondar, Ethiopia

**Keywords:** Knowledge, Practice, Iodized salt, Reproductive age, Ethiopia

## Abstract

**Objective:**

The objective of this study was to assess knowledge and practice of iodized salt utilization among reproductive women in Addis Ababa city. A cross-sectional study was carried out on 549 households. A sample district was designated by using the simple random sampling techniques. Data were collected by a face-to-face interview and household salt was tested to check whether its practice was good. p < 0.2 in the bivariate logistic regression was entered into the multivariable logistic regression, and p < 0.05 was considered as significantly associated.

**Results:**

Mothers who had good knowledge and practice of iodized salt were 78% (95% CI 74.9, 81.2) and 76.3% (95% CI 72.7, 79.8), respectively. Monthly household income (AOR = 2.97; 95% CI 1.20, 7.37) was associated with knowledge of iodized salt of respondents. Similarly, educational status (AOR = 2.45; 95% CL 2.10, 6.43) of respondents was significantly associated with the practice of iodized salt. This study indicated that increasing the level of knowledge and practice of iodized salt was good. Monthly household income and educational status were associated with knowledge and practices of iodized salt of respondents. Hence, improving mothers’ education is a highly recommended strategy for addressing public health problems of iodine deficiency.

## Introduction

Iodine is an essential dietary nutrient for the thyroid hormones that regulate the growth and development of humans and animals. It plays an important role in controlling body metabolic rate, growth and development of body structures by producing the thyroid hormone [1, 2]. According to the World Health Organization (WHO), adults need about 120 μg of iodine per day to prevent iodine deficiency disorders (IDD) [3].

Poor intake of iodine is the major public health problem of women in the reproductive age pregnant and lactating women are particularly the most susceptible to iodine deficiencies which expose them to getting irreversible mentally impaired babies [2]. It is also documented that it causes abortions, stillbirths, congenital abnormalities, cretinism, goiter and impaired mental function as well as squinting, hypothyroidism, and stunting [4, 5]. Moreover, it impedes learning capacity, women’s health, the quality of life of communities, and the economic productivity of nations [4].

Globally, 38% of the world’s population lives with insufficient iodine [6]. Africa with its 9321.1 million deficient people bears the most burden of the region [7]. In Ethiopia, 35 million people are at risk of iodine deficiency, and the 50,000 annual prenatal deaths are related to this problem [8]. As a result, universal salt iodization (USI) is recommended as the most cost-effective, safe and sustainable strategy to eliminate IDDs [9]. Fortunately, 76% of households are consuming adequate iodized salt globally [10].

Regional coverages of iodized salt vary from 90% in Asia and the Pacific region to 40–60% in Sub-saharan Africa [10]. The level of utilization also varies from 10 to 90% in different countries. For instance, the utilization of iodized salt is less than 10% in Sudan, Mauritania, Guinea-Bissau, and Gambia, whereas Burundi, Kenya, Nigeria, Tunisia, Uganda, and Zimbabwe have achieved the USI target [7].

In Ethiopia, the practice of adequate iodized salt use showed a marked increase from 15% in 2011 to 89% in 2016 [11, 12]. Inconsistencies of the practice are detected among dwellings and economic status. As an illustration, iodized salt utilization is the highest in Tigray (55.2%) and Somali (49.4%) and the lowest in Gambela (9.5%), SNNPR (13.7%), and Amhara regions (15%) [13]. As a result, 10.8–36% [14, 15] of women aged 15–49 years have been affected by goiter.

The Government of Ethiopia revitalized and launched universal salt iodization initiatives and planned strategies for the achievement of a virtual elimination of iodine deficiency disorders through achieving universal salt iodization [16, 17]. However, still, only 26% of the households are using adequate iodized salt [14]. In fact, IDD stays the major public health problem among all segments of the population [14, 18]. Besides, there is limited information on knowledge and practice of iodized salt use in Addis Ababa. Therefore, this study aimed to assess the knowledge and practice of iodized salt utilization among reproductive women in the city.

## Main text

### Methods

Community based cross-sectional study design was conducted to assess Knowledge and Practice on iodized salt among reproductive age group women in Yeka Sub City Addis Ababa. Addis Ababa is a Capital City of Ethiopia. The City has 10 boroughs named sub-Cities and 99 districts. Yeka is one of the 10 sub cities of Addis Ababa, with the total population of a 413, 175. The sub-City has 13 districts [19].

All the women in the reproductive age in Addis Ababa were used as the source of the population this study. The sample size was calculated by using a single proportion formula through by judging the following assumptions; 29.6% as prevalence of salt practice [11], 95% confidence level, and 4% degree of precision. Finally, the sample size of 550 was obtained by considering 10% non-response rate. A multistage stratified sampling technique followed by systematic sampling technique was employed to select study participants. Four districts were selected by lottery method among 13 districts in Yeka sub-city. Then, the total sample size was allocated proportionally to each selected district. Finally, Households were selected using systematic random sampling technique.

Structured interviewed and observation technique was used to collect data. The questionnaire was first prepared in English and then translated into Amharic. The questionnaire was developed by through by different literature review, other similar studies and EDHS [11, 20–22]. Two day training was given for data collectors and supervisors who have extensive experience in data collection about the methods of interview and observation. A total of 6 clinical nurses as data collector and 2 public health experts as supervisor were recruited for the study. During the data collection, close supervision was done by the principal investigator and supervisors.

The knowledge of the respondents towards iodized salt use were computed by using eight knowledge item questions accordingly, participants who respond correctly to knowledge questions and score median and above the median value considered as good knowledge whereas, respondents respond below median value were supposed as poor knowledge. Practice of iodized salt was summarized by applying four practice questions, as a result, participants who respond correctly answered practice question score median and above the median value considered as good practice whereas, respondents respond below median value were supposed as poor practice of iodized salt. Concerning media exposure women who read a newspaper or magazine or listen to the radio, or watched television at least once per month were considered having satisfactory media exposure and salt iodine content estimation was done by using a rapid test kit (RTK). The colour of the test sample is compared with the standard colour chart for calculating the salt iodine content. Finally, salt with ≥ 15 PPM was categorized as adequately iodized salt, whereas < 15 PPM was considered non-iodized salt.

All returned questionnaire were checked for completeness and consistency of responses manually. The data were entered using Epi-info version 3.5.4. Analysis associations between dependent and independent variables was assessed by using binary logistic regression and variables with p value < 0.2 entered into multiple logistic regression with 95% Confidence Intervals. Corresponding p value of < 0.05 was considered as statistically significant at 95% of confidence interval.

### Result

Nearly half (45.5%) of the respondents were in the age group of 36–49 years; two-thirds (62.7%) were married and 25.5% of the respondents had university degrees. About 35.8% were employed, while 25.3% of the households earned a monthly income of less than ETB 1500. The majority (69.9%) of the husbands were employed of whom 10.2% were unable to read and write. A huge proportion (88.3%) of the respondents indicated that they had heard about iodized salt. Radio and television were the major media for 73.3% of the information about the importance of iodized salt and iodine-deficiency diseases. They said that a legal frame which prohibits the production, trade, and sales of non-iodized salt is in place in Ethiopia. Nearly two-thirds (60.8%) of the respondents added salt at the end of cooking. The majority (88.9%) of the respondents stored their salt in closed containers. Some (33.5%) participants consumed salt with an iodine level of ≥ 15 ppm (Table 1).

**Table 1:** Reproductive age women and their husbands Socio-demographic characteristics in Yeka sub-city, Addis Ababa, March 2015

Variable	Frequency	Percent (%)
Age of the respondents		
15–25	76	13.8
26–35	223	40.7
36–49	250	45.5
Religious of the respondents		
Orthodox	372	67.8
Protestant	86	15.7
Muslim	79	14.4
Catholic	11	2.0
Other	1	0.2
Ethnicity of the respondents		
Amhara	240	43.7
Oromo	151	27.5
Gurage	85	15.5
Tigire	56	10.2
Other	17	3.1
Marital status		
Married	344	62.7
Unmarried	205	37.3
Educational status of the mother	72	13.1
Unable to read and write	49	8.9
Read and write	23	4.2
Primary education	137	25
Secondary education	156	28.4
Vocational Diploma	44	8.0
Degree and above	140	25.5
Respondents occupational		
Own business	138	25.1
House wife	128	23.3
Private employee	101	18.4
Government employee	79	14.4
Unemployed	40	7.3
Daily laborer	38	6.9
Other	25	4.6
Household monthly income (ETB)^a^		
< 1500	239	25.3
1501–2800	139	25.3
2801–5000	164	29.9
> 5000	107	19.5
Age of the husband (n = 352)		
23–35	104	29.5
36–50	176	50.0
> 50	72	20.5
Religion of the husband (n = 352)		
Orthodox	229	65.1
Muslim	57	16.2
Protestant	49	13.9
Catholic	14	4.0
Other	1	0.4
Husband educational status (n = 352)		
Unable to read and write	32	9.1
Read and write	4	1.1
Primary education	37	10.5
Secondary education	100	28.4
Vocational diploma	27	7.7
University degree	152	43.2
Husband occupation		
Private employee	136	38.6
Government employee	90	25.6
Own business	76	21.6
Non-governmental organization	20	5.7
Daily laborer	18	5.1
Other	12	3.4
Have you heard about iodized salt		
Yes	485	88.3
No	64	11.7
The source of information about iodized salt		
Radio, television	402	73.2
Printed material	68	12.4
Friends/neighbors	26	4.7
Health workers	20	3.7
Others	2	0.4
Have you heard the effects of iodine deficiency on human		
Yes	436	79.4
No	113	20.6
Iodized salt should be handled in the store and household with great care than non-iodized		
Yes	86	15.7
No	463	84.3
What are the advantage of using iodized salt?		
Better test	24	4.4
Better digestion	3	0.5
Makeup for iodine in the human body (prevent IDD such as goiter, abortion)	401	73
I don’t know	99	18
Other	22	4
What is the consequence of Iodine deficiency?		
Goiter	420	76.5
Cretinism/mental retardation in children	118	21.5
Abortion/still birth/miscarriage	96	17.5
Regular consumption of iodized salt can remove iodine deficiency in the body?		
Yes	408	74.3
No	141	25.7
Health risk of unborn baby if there is lack of iodine in the diet of pregnant women?		
Risk of being mentally impaired	170	31
Risk of being physically impaired	68	12.4
I don’t know	270	49.5
Other	39	7.1
Group of population should receive much iodine than other		
Children	220	40.1
Pregnant women	179	32.6
All people need same amount	195	35.5
I don’t know	92	16
Other	4	0.7
Is there legal frame exist in Ethiopia which prohibit production, trade and sales of non-iodized salt?		
Yes	70	12.8
No	246	44.8
I don’t know	233	42.4
Does iodine in the salt affect its test?		
Yes	75	13.7
No	329	59.9
I don’t know	145	26.4
In what place should iodized salt be kept		
Dry place	235	42.8
With no direct sunlight contact	71	12.9
In closed container	358	65.3
It doesn’t need special place	31	5.6
I don’t know		
Timing of adding salt during food cooking process		
At the beginning	45	8.4
In the middle	168	30.8
At the end	334	60.8
Where do you usually store your salt		
In an open package	25	4.6
In container without lid	36	6.5
In container with closed lid	488	88.9
Iodine test result		
Not iodized (0 ppm)	160	29.1
Inadequate iodine in the salt (< 15 ppm)	200	36.4
Adequate iodine in the salt (> 15 ppm)	184	33.5
No salt at home	5	0.9
Place where salt is store		
Exposed to sun light	4	0.7
Near to fire in the kitchen	7	1.3
Far from sun light and fire	536	97.6
Other	2	0.4

^a^Indicate One US Dollar = 27.00 Ethiopian Birr (ETB)

The overall prevalence of knowledge and practice of iodized salt use among women in reproductive age in Addis Ababa was 78% (95% CI 74.9, 81.2) and 76.3% (95% CI 72.7, 79.8), respectively.

Women living on a monthly household income greater than ETB 5000 had 2.97 times better knowledge on iodized salt use [AOR: 2. 97; 95% CI (1.20, 7.37)] compared to those who lived on a monthly income of less than ETB1500 (Table 2). Women who had college and above educational status were 2.45 (2.10, 6.43) times more likely to use iodized salt compared to mothers unable to read and write. Similarly, the odds of having earning a monthly household income greater than ETB 5000 were 3.66 times [AOR: 3.66; 95% CI (1.78, 8.03)] higher among respondents who had good practice of iodized salt use compared to their counterparts (Table 3).

**Table 2:** Bivariate and multivariable logistic regression output showing that factors associated with knowledge of iodized salt among women at reproductive age, in Yeka Sub-city Addis Ababa, March 2015

Variables	Knowledge		Crude Odds Ratio with 95% CI	Adjusted Odds Ratio with 95% CI
	Good	Poor		
Respondents educational status				
Unable to read and write	33 (67.3)	16 (32.7)	1	1
Read and write	19 (82.6)	4 (17.4)	2.30 (0.67,7.90)	2.44 (0.68,8.68)
Primary education	84 (61.3)	53 (38.7)	0.77 (0.39,1.53)	0.76 (0.37,1.55)
Secondary education	128 (82.1)	28 (17.9)	2.22 (1.08,4.57)	2.02 (0.92,4.44)
Degree and above	164 (89.1)	20 (10.9)	3.98 (1.87,8.47)	2.72 (1.05,7.03)
Occupations of the respondents				
Government employee	69 (87.3)	10 (12.7)	1.08 (0.34,3.41)	1.21 (0.35,4.13)
Private employee	87 (86.1)	14 (13.9)	0.97 (0.32,2.91)	1.64 (0.51,5.27)
Own business	101 (73.2)	37 (26.8)	0.43 (0.16,1.18)	1.02 (0.33,3.14)
House wife	88 (68.8)	40 (31.2)	0.34 (0.13,0.95)	0.97 (0.30,3.06)
Unemployed	27 (77.1)	8 (22.9)	0.53 (0.15,1.80)	1.01 (0.47,2.10)
Daily laborer	24 (77.4)	7 (22.6)	0.54 (0.15,1.90)	1.19 (0.32.4.51)
Others	32 (86.5)	5 (13.5)	1	1
Household monthly income				
< 1500	98 (70.5)	41 (29.5)	1	1
1500–2800	100 (71.9)	39 (28.1)	1.07 (0.64,1.80)	0.85 (0.48,1.49)
2801–5000	131 (79.9)	33 (20.1)	1.67 (0.98,2.82)	0.97 (0.54,1.83)
> 5000	99 (92.5)	8 (7.5)	5.18 (2.31,11.61)	2.97 (1.20,7.37)*
Marital status				
Not married	164 (80)	41 (20)	1	1
Married	264 (76.7)	80 (23.3)	2.59 (0.81,8.32)	0.86 (0.53,1.40)
Media exposure				
Poor	7 (58.3)	5 (41.7)	1	1
Good	421 (78.4)	116 (21.6)	1.21 (0.79,1.85)	1.15 (0.33,3.94)

* Indicate significant at p value less than 0.05 in multivariable logistic analysis

**Table 3:** Bivariate and multivariable logistic regression output showing that factors associated with practice of iodized salt among women at reproductive age, in Yeka Sub-city Addis Ababa, March 2015

Variables	Practice		Crude Odds Ratio with 95% CI	Adjusted Odds Ratio with 95% CI
	Good	Poor		
Respondents educational status				
Unable to read and write	19 (38.8)	30 (61.2)	1	1
Read and write	12 (52.2)	11 (47.8)	0.04 (0.02,0.11)	1.18 (0.38,3.64)
Primary education	86 (62.8)	51 (37.2)	0.08 (0.03,0.22)	2.06 (0.98,4.32)
Secondary education	131 (84)	25 (16)	0.12 (0.05,0.25)	2.10 (1.24,5.6)
Technical/vocational	40 (90.9)	4 (9.1)	0.36 (0.16,0.80)	2.14 (1.55,5.43)
Degree and above	101 (93.6)	9 (6.4)	0.69 (0.20,2.35)	2.45 (2.10, 6.43)*
Occupations of the respondents				
Government employee	75 (94.9)	4 (5.1)	2.93 (0.74,11.63)	2.85 (0.64,12.76)
Private employee	83 (82.2)	18 (17.8)	0.72 (0.25,2.10)	1.07 (0.32,3.57)
Own business	98 (71)	40 (29)	0.35 (0.14,1.05)	1.08 (0.31,3.51)
House wife	95 (74.2)	33 (25.8)	0.45 (0.16,1.25)	1.41 (0.41,4.79)
Unemployed	31 (88.6)	4 (11.4)	1.21 (0.30,4.93)	3.53 (0.75,16.07)
Daily laborer	5 (16.1)	26 (83.9)	0.03 (0.08,0.12)	0.56 (0.25,1.51)
Others	32 (86.5)	5 (13.5)	1	
Household monthly income				
< 1500	84 (60.4)	55 (39.6)	1	1
1500–2800	94 (67.6)	45 (32.4)	1.37 (0.84,2.24)	0.98 (0.50,1.60)
2801–5000	138 (84.1)	26 (15.9)	3.48 (2.03,5.96)	1.69 (0.87,3.27)
> 5000	103 (96.3)	4 (3.7)	2.57 (1.50,7.23)	3.66 (1.78,8.03)*
Marital status				
Not married	146 (71.2)	59 (28.8)	1	1
Married	273 (79.4)	71 (20.6)	1.55 (1.04,2.32)	1.30 (0.77,2.18)
Media exposure				
Poor	5 (41.7)	7 (58.3)	1	1
Good	414 (77.1)	123 (22.9)	4.71 (1.47,15.11)	1.52 (0.44,5.27)
Knowledge				
Poor	81 (66.9)	40 (33.1)	1	1
Good	338 (79)	90 (21)	1.86 (1.19,2.890)	1.27 (0.75,2.14)

* Indicate significant at p value less than 0.05 in multivariable logistic analysis

### Discussion

It is apparent that the use of iodized salt by individuals and households is the major approach in the control of IDDs globally [23–25]. Thus, strengthening salt iodization programs and improving monitoring is a crucial step to eradicate the problem [22, 26, 27]. This study found that 78% of the respondents had good knowledge of iodized salt use. The finding was higher than the 26% reported from Tehran [22]. The variation might be due to the nature of study settings in that woman from slum areas and poor communities were included in the Tehran study. Clearly, slum areas are characterized by poor infrastructure and inadequate communication channels compared to Addis Ababa, the setting of our study, where the respondents had ample opportunities to increase their knowledge through promotions of iodized salt on the media. In fact, promotions on the media increase public awareness and alert that all salt producers and traders duly iodize their salt which is essential for achieving the USI goal [18, 28]. Our finding was slightly lower than 90.4% reported in Ghana [5]. This might be due to the accessibility of different media nearby for the target group in the study area.

Regarding the practice of iodized salt use of reproductive women, this finding is also higher than the 14% noted in Tehran [22]. The difference could be due to the fact that our study was used to ascertain the outcome by using two cut-off points, whereas the Tehran study considered three classifications as to determine outcome variable.

Household monthly income of the respondents was one of the factors associated with knowledge of iodized salt use of reproductive women. Accordingly, women belonging to greater than ETB 5000 monthly income were about three times more likely to have good knowledge of iodized salt compared to women belonging to less than ETB 1500 monthly household income group. This might be because women who lived on higher socioeconomic status had chances to purchase and use different electronic equipment which is important for enhancing nutrition education. In addition, house-to-house health visits by urban health workers improves knowledge of iodized salt utilization [29]. Besides, household income can be strongly associated with the type of salt used. That is, poorer households are much more likely to consume coarser salt owing to their low purchasing power [30].

The odds of practicing iodized salt were 2.45 times higher among reproductive age women who were university degree and above graduates compared to those who were unable to read and write. This finding is supported by those of studies done in Wolaita [31] and Arsi [28] zones. This might be due to the fact that higher levels of education provided better nutritional awareness about the benefits of iodine, increased awareness on the health benefits of iodine in diets, and raised the use of iodized salt. In addition, women who had the highest educational status had good employment opportunities which might be indicative of better socioeconomic status. This could relate to women who were better educated and had the ability of purchasing good quality food appropriate for salt iodization practice [30]. Moreover, mothers who were less educated and had less resources, had the least knowledge about the importance of iodized salt [20].

Finally, the probabilities of practicing iodized salt use were high among reproductive women who had higher monthly income compared to those who had low income. This finding was supported by researches elsewhere [24, 28]. Confirming the fact that better practices of iodized salt by women in the reproductive age has a relationship to the price of the salt. Women who earned better monthly household income have the ability of purchasing and utilizing iodized salts for high prices [32]. Similarly, household income might be strongly associated with the types of salt preference in that poorer households were much more likely to purchase coarser salts [30]. Furthermore, excess cost of iodized salt might be a barrier to preventing iodine deficiency because it forces people with low incomes not to buy and use the salt [29, 33]. Thus, most women may not able to use it due to its high cost [21].

In conclusion, this study showed that women had good levels of knowledge and practice of iodized salt use. Monthly household income and educational status are associated with the knowledge and practices of iodized salt of respondents. Hence, improving mothers’ education is an important strategy to address the public health problems of IDDs.

## Limitation of the study

Rapid test kit show only color change which cannot tell the exact amount of iodine concentration in the salt but due to resource constraint gold standard iodine test couldn’t be use which show the exact concentration. This study didn’t triangulate with qualitative study.

## Abbreviations

ACIPH: Addis Continental Institute of Public Health
AOR: Adjusted Odd Ratio
CI: confidence interval
COR: Crude Odd Ratio
IDD: iodine deficiency disorder
UNICEF: United Nations International Children’s Education Fund
USI: universal salt iodization
PPM: part per million
SPSS: Statically Package for Social Science
WHO: World Health Organization
